# Supplementary material for: Cell-Int: a cell–cell interaction assay to identify native membrane protein interactions
Source: Life Sci Alliance. 2024 Sep 5;7(11):e202402844. doi: 10.26508/lsa.202402844 (PMC11377309; doi:10.26508/lsa.202402844)
Supplement: Supplementary file 1 [file LSA-2024-02844_TableS1.docx]

**Table S1.** **IC50 values of inhibitory antibodies tested in this study and comparison to previous data**

| **Antibody** | **Interacting pair** | **IC50 values (µg/mL)** | | **Reference** |
| --- | --- | --- | --- | --- |
|  |  | **This study** | **Previous studies** |  |
| Nivolumab | PD-1/PD-L1 | 1.18 | 0.36 | Wang et al., 2014 |
|  | PD-1/PD-L2 | 0.99 | 0.37 | Wang et al., 2014 |
| Atezolizumab | PD-1/PD-L1 | 1.62 | 0.29 | Li et al., 2021 |
| Cetuximab | EGF/EGFR | ND | 0.027 to 0.165 | Bovio et al., 2020 |
|  | H-EGF/EGFR | 1.81 | ND | N/A |
| Anti-LILRB1 20G10 | HLA-G1/LILRB1 | 5.47 | ND | N/A |

ND: not determined; N/A: not applicable
